# Research progress on the characteristic features of non-suicidal self-injury addiction, neuroimaging correlates, and evidence-based interventions in adolescents

**DOI:** 10.3389/fpsyg.2026.1704139

**Published:** 2026-02-20

**Authors:** Weiwen Chen, Yinan Zhuo

**Affiliations:** 1 The Second Affiliated Hospital of Guangdong Medical University, Zhanjiang, China; 2 Beijing Normal University, Beijing, China

**Keywords:** adolescence, behavioral addiction, DBT, emotion dysregulation, fronto-limbic circuitry, neurobiology, non-suicidal self-injury, risk and protective factors

## Abstract

**Background:**

Non-suicidal self-injury (NSSI) represents a significant public health concern, particularly among adolescents. Recent conceptualizations posit that a severe, repetitive form of NSSI may be understood through an addiction framework, characterized by craving, loss of control, and continued use despite harm. However, an integrative model linking its behavioral phenotype, neurobiological underpinnings, and evidence-based treatment remains to be fully articulated.

**Methods:**

This narrative review synthesizes contemporary literature across psychological, neurobiological, and clinical domains to propose an integrative model of NSSI with addictive features.

**Results:**

The review establishes three key pillars of evidence. First, behaviorally, repetitive NSSI exhibits core addiction-like features driven by potent negative reinforcement. Intriguingly, this occurs alongside intact or even enhanced reactive inhibitory control, suggesting a specific dysregulation in reward-based decision-making rather than a global impulse deficit. Second, neurobiologically, this phenotype is supported by convergent findings of hypothalamic–pituitary–adrenal (HPA) axis dysregulation, a reward system biased towards distress relief, and dysfunctional fronto-limbic circuitry marked by limbic hyperreactivity and impaired prefrontal regulation. Third, therapeutically, Dialectical Behavior Therapy (DBT) emerges as the most effective intervention, with emerging evidence indicating it promotes symptom reduction through the neuroplastic normalization of this same fronto-limbic circuitry. The development of this addictive cycle is moderated by a transaction between risk factors (e.g., childhood adversity, peer victimization) and protective factors (e.g., resilience, self-efficacy).

**Conclusion:**

This synthesis supports the heuristic value of an addiction model for severe NSSI, providing a coherent framework that bridges behavior, brain, and treatment. Future translational research should focus on identifying biomarkers for the addictive subtype, developing circuit-targeted interventions, and implementing staged, multi-systemic prevention strategies.

## 1 Introduction

Non-suicidal self-injury (NSSI) refers to the intentional harm inflicted on body tissue without suicidal intentions, using methods that deviate from societal norms (Halicka and Kiejna, 2018). This spectrum of behaviors includes cutting, burning, and severe scratching or an intentional overdose of a substance with the aim of achieving a desired change through actual or anticipated physiological consequences, and is not fatal (Plener et al., 2018; McEvoy et al., 2023). Epidemiological studies show that self-harm among adolescents is prevalent and widespread globally, with reported prevalence rates ranging from 16.8 to 44.8% across different countries. Non-suicidal self-harm is significantly associated with suicide risk, and there are marked gender and geographical differences in this association (Lim et al., 2019; Faura-Garcia et al., 2022; Poudel et al., 2022; Shi et al., 2025). Non-suicidal self-harm (NSSI) among Chinese adolescents is characterized by high incidence and group specificity. Nationwide data suggests a high overall prevalence (22.37%), with left-behind children being a particularly high-risk group. Family environment and psychological factors (such as family conflict and depression) play a crucial role in its occurrence and development (Lang and Yao, 2018; Liu et al., 2025; Zheng et al., 2025).

A significant development in the classification of NSSI is its growing acknowledgment as a separate clinical syndrome, rather than merely a symptom of broader psychopathology like borderline personality disorder. This change is officially documented in the Diagnostic and Statistical Manual of Mental Disorders, Fifth Edition (DSM-5), where “Non-suicidal Self-Injury Disorder” is placed in Section III for conditions requiring additional research, recognizing its distinct phenomenological features and developmental path (American Psychiatric Association, 2013).

A significant obstacle to effective treatment is the frequent presence of addictive traits in many cases. These traits include persistence, resistance to change, and continued behavior despite negative outcomes, which are characteristic of addictive behaviors. However, the neurobiological basis of NSSI, especially the mechanisms that contribute to its addictive nature, is not fully understood. This review aims to summarize recent progress in three main areas: the addiction-like features of NSSI, its neurobiological underpinnings focusing on findings from neuroimaging studies, and the current landscape of evidence-based treatments. This synthesis seeks to guide the development of more targeted and efficient interventions for this at-risk population.

## 2 Addiction-like phenotype and psychological mechanisms of NSSI

NSSI often serves as a maladaptive regulatory strategy in response to acute negative affective states, such as depression and anxiety, triggered by stressful events. A defining characteristic of repetitive NSSI is the subjective experience of an “inability to resist the urge to self-harm,” a description that has influenced its conceptualization within an addiction framework. The behavioral cycle is sustained through operant reinforcement mechanisms: the act of self-injury leads to immediate relief from aversive emotional states, representing negative reinforcement, and may, in some cases, produce a sense of relief or calm, signifying positive reinforcement (Nock and Prinstein, 2004; Blasco-Fontecilla et al., 2016; Turner et al., 2019). This reinforcement model resembles that found in substance use disorders, creating a behavioral dependency in which individuals may increase the frequency or intensity of NSSI to achieve the desired psychophysiological effect, develop tolerance, and continue the behavior despite clearly harmful consequences (Blasco-Fontecilla et al., 2016; Shu et al., 2025; Stevens, 2015).

Contemporary cognitive research necessitates a reevaluation of earlier reductionist models that equated NSSI primarily with global deficits in impulse control. In contrast to these postulates, Mirabella and colleagues found that adolescents diagnosed with NSSI disorder demonstrated enhanced reactive inhibitory control compared to their typically developing peers (Mirabella et al., 2024). This finding supports a theoretical perspective suggesting that the behavior may not stem from an inability to inhibit motor responses, but rather from the strong negative reinforcement associated with distress reduction through self-injury, a notion previously proposed by Fikke et al. (2011).

The psychosocial origins of NSSI are multifaceted. Among Chinese adolescents, adverse childhood experiences have been identified as a significant risk factor, which can increase the risk of NSSI both directly and indirectly through its impact on psychological sub-health (Huang et al., 2022). The key motivations for initiating NSSI are diverse. Meta-analytic evidence indicates that intrapersonal functions, particularly emotion regulation (reported by 63–78% of individuals), are most prevalent, while interpersonal functions (e.g., expressing distress) are also common (33–56%) (Taylor et al., 2018). Over time, the behavioral development of NSSI can parallel pathways observed in substance addiction, advancing from an initial, reward-based (e.g., distress relief) and motivated behavior to a compulsive, habitual, and routine-driven pattern that persists even when the original motivations are less salient (Zhu et al., 2024; Carenys and Adan, 2025). This transition from a goal-directed to a habitual behavior underscores its addictive potential.

## 3 Neurobiological substrates of NSSI addiction

Empirical evidence supports the neurobiological plausibility of addictive processes in NSSI, situating it within a framework that shares fundamental mechanisms with substance and behavioral addictions. This evidence encompasses molecular, neurochemical, and systems neuroscience levels.

### 3.1 Neurochemical and neuroendocrine dysregulation

At the molecular level, self-injury is believed to initiate the release of endogenous opioids, which may contribute to affect regulation (Bresin and Gordon, 2013). This response is associated with an analgesic effect that alleviates pain, a finding supported by studies on reduced pain sensitivity in NSSI (Franklin et al., 2012; Boyne and Hamza, 2022). Over time, repeated engagement may lead to neuroadaptive changes similar to those in substance dependence, potentially involving the endogenous opioid system and resulting in physiological tolerance. Consequently, individuals may need to engage in more frequent or severe self-injury to attain the same relief, thereby perpetuating an addictive cycle, as suggested by reviews of the addictive model of NSSI (Carenys and Adan, 2025).

The reward-dysregulation model of NSSI is further supported by evidence pointing to potential dysfunction within the body’s primary stress-response system. Neuroendocrine research has investigated the role of the hypothalamic–pituitary–adrenal (HPA) axis, with some studies linking NSSI to indicators of chronic stress burden, such as elevated basal cortisol levels and a blunted cortisol awakening response (Kaess et al., 2012). However, a recent comprehensive narrative review highlights that the relationship between cortisol levels and NSSI is complex and not yet fully consistent across studies, with findings showing mixed results likely due to methodological variations and sample characteristics (Sporniak and Szewczuk-Boguslawska, 2025). Despite this heterogeneity, the theoretical significance of HPA axis dysregulation remains compelling. It is hypothesized that when present, such dysregulation may lower an individual’s threshold for tolerating distress and amplify negative affect. According to the experiential avoidance model (Nock, 2009), this heightened state of aversive arousal can, in turn, increase the drive to engage in NSSI as a rapid, albeit maladaptive, strategy to achieve immediate relief.

### 3.2 Structural and functional neural circuitry alterations

Neuroimaging studies have identified consistent abnormalities in neural circuits that support emotion processing, reward valuation, and inhibitory control—functions that are significantly disrupted in addictive disorders (Auerbach et al., 2021).

Neuroimaging studies suggest that aberrant reinforcement processing may underpin NSSI. On one hand, individuals with NSSI show heightened neural sensitivity to rewards. For instance, adolescents with thoughts of NSSI exhibited increased activation in the putamen, a core reward-processing region, in response to monetary gains (Poon et al., 2019). On the other hand, they may have deficits in learning from punishment. A recent fMRI study found that young adults with NSSI were less accurate at avoiding punishing options and showed reduced activation in the nucleus accumbens (NAcc) during punishment avoidance compared to reward selection (Nicolaou et al., 2025). This combination of heightened reward reactivity and blunted punishment sensitivity could disrupt adaptive decision-making.

Complementing these subcortical alterations, evidence points to functional and structural deficits in prefrontal regulatory regions critical for cognitive control and emotion regulation. Neuroimaging reviews highlight the involvement of the dorsolateral and ventrolateral prefrontal cortices (dlPFC, vlPFC) and the anterior cingulate cortex (ACC) in both suicidal and non-suicidal self-injurious behaviors (Auerbach et al., 2021; Schmaal et al., 2020). Of note, structural studies have specifically reported reduced ACC volume in youth with NSSI (Auerbach et al., 2021). These prefrontal abnormalities may underlie a diminished capacity for cognitive reappraisal and impulse inhibition.

The pathology also involves impaired communication within the fronto-limbic circuit. Studies using multimodal fMRI have identified widespread amygdala circuitry anomalies in adolescents with NSSI, including atypical amygdala-frontal connectivity (Schreiner et al., 2017). Such disrupted connectivity, particularly decreased inhibitory control from prefrontal regions over limbic structures like the amygdala, is a consistent finding in models of dysregulated behavior (Schmaal et al., 2020).

This compromised top-down control may permit unregulated, limbic-driven impulses. Recent evidence directly supports this, showing that diminished spontaneous activity in the left middle frontal gyrus—a key prefrontal node—is strongly associated with the severity of suicidal ideation in adolescents with NSSI (Li et al., 2025). Furthermore, the reward system itself appears to be reconfigured in NSSI with addictive features. A resting-state fMRI study comparing adolescents with addictive versus non-addictive NSSI found altered functional connectivity of key reward regions, including reduced connectivity between the nucleus accumbens (a core part of the ventral striatum) and the medial cingulate cortex (Liao et al., 2025). This pattern of dysconnectivity within reward and emotion-processing circuits supports the view that the reward system may be reoriented towards a maladaptive pattern of negative reinforcement in addictive NSSI.

### 3.3 A synthesized neurobiological model

A synthesized neurobiological model of NSSI addiction emerges from the interplay of various factors. A predisposing or acquired dysregulation of the hypothalamic–pituitary–adrenal (HPA) axis heightens stress sensitivity and negative affectivity (Kaess et al., 2012). In response to distress, hyperreactive limbic structures, such as the amygdala and insula, generate intense aversive emotional states (Auerbach et al., 2021). At the same time, impairments in executive functions—which are closely linked to prefrontal regulation—impair the ability to regulate these emotional states through adaptive cognitive strategies (Fikke et al., 2011; Auerbach et al., 2021). The act of self-injury offers a potent, albeit harmful, “short-circuit” solution by triggering an endogenous opioid and dopaminergic response that temporarily reduces limbic activity and alleviates distress (Wu et al., 2023). This strong negative reinforcement, occurring within a context of disrupted reward signaling, reinforces the maladaptive behavior loop (Nock and Prinstein, 2004). Over time, neuroadaptations foster tolerance and habituation, entrenching the individual in an addictive cycle that becomes ingrained in the very architecture and chemistry of their brain (Luo et al., 2024). It is important to interpret this model with caution.

It is important to interpret this model with caution, acknowledging key limitations: (1) Causality and Directionality: the predominance of cross-sectional neuroimaging data limits definitive causal conclusions. The directional language used in this model (e.g., circuit dysfunction “contributing to” symptoms) should be understood as a heuristic framework for generating testable hypotheses, rather than an established causal pathway. (2) Reverse Inference: it is equally plausible that the observed neural correlates (e.g., amygdala hyperreactivity, prefrontal deficits) represent the biological sequelae of chronic developmental stress or repeated trauma, rather than the primary drivers of the addictive cycle. This reverse inference problem underscores the critical need for longitudinal research to disentangle cause from consequence.

The frequent comorbidity of NSSI with other psychiatric conditions highlights the non-specificity of its underlying neural correlates. Meta-analytic evidence confirms a significant co-occurrence of NSSI with suicidal behavior, with a pooled prevalence of 9.6% in mixed populations and rising to 26% among individuals with mental disorders (Ye et al., 2022). Epidemiological studies further reveal a profound bidirectional link, where a substantial proportion of individuals with NSSI also report suicidal behaviors, and vice versa (Voss et al., 2020). This pattern of comorbidity extends beyond affective and impulse-control disorders to include conditions like disordered eating, underscoring its transdiagnostic nature (Eisenman et al., 2025).

## 4 Evidence-based interventions for NSSI

### 4.1 Pharmacotherapeutic approaches

Currently, no pharmacologic agent has received regulatory approval for the specific indication of NSSI (Witt et al., 2021). Consequently, clinical practice relies on agents that target co-occurring symptoms, such as depression or anxiety. While medications including selective serotonin reuptake inhibitors (SSRIs), naltrexone, and atypical antipsychotics are sometimes used adjunctively, a contemporary narrative review on pharmacological support emphasizes that their role is supportive, the evidence base remains limited and requires validation, and their use is often restricted by side effects (Rogalski and Tomczak, 2025). Notably, there is concern that SSRIs may even exacerbate NSSI risk in the initial months of treatment for adolescent depression (Xu M. et al., 2025; Xu Y. et al., 2025; Chen et al., 2025). Therefore, a substantial and consistent evidence base confirming the efficacy of pharmacotherapy for the core symptoms of NSSI is still lacking, underscoring the primacy of evidence-based psychotherapeutic interventions.

### 4.2 Psychotherapeutic intervention: dialectical behavior therapy

A recent and comprehensive network meta-analysis of treatments for adolescent NSSI concluded that psychotherapy, particularly dialectical behavior therapy (DBT), demonstrates superior efficacy compared to pharmacotherapy (Chen et al., 2025). DBT’s therapeutic framework specifically targets the emotional dysregulation and behavioral disinhibition central to NSSI, employing a multimodal protocol that includes individual psychotherapy, group skills training, intersession coaching, and therapist consultation teams (Dallenbach et al., 2025).

The efficacy of DBT in reducing self-injurious behaviors among youth is well-established. Meta-analytic findings confirm that DBT significantly decreases self-directed violence, a category encompassing both suicide attempts and NSSI (DeCou et al., 2019). This efficacy is robustly demonstrated in adolescent populations. A landmark randomized clinical trial with adolescents at high risk for suicide found that DBT outperformed supportive therapy in reducing suicide attempts, NSSI, and overall self-harm during the active treatment phase (McCauley et al., 2018). The difficulty in reducing suicide reattempts with brief or non-specialized interventions—illustrated by a recent trial where a brief adjunctive program showed no significant added benefit over treatment as usual (Garcia-Fernandez et al., 2025)—further underscores the value of comprehensive, skills-based approaches like DBT.

Collectively, this evidence indicates that DBT leads to significant reductions in the frequency and severity of NSSI and suicidal behavior, while enhancing emotion regulation skills in adolescents. It is important to note that this robust evidence base derives largely from youth with high acuity and co-occurring disorders (e.g., borderline personality disorder), establishing its efficacy for severe presentations. Further research is needed to optimize interventions for adolescents with NSSI in less specialized settings.

DBT utilizes a dialectical framework integrating acceptance-oriented strategies with skills training for behavioral change, including modules on mindfulness, distress tolerance, emotion regulation, and interpersonal effectiveness. A core component is the group skills training segment (DBT-ST). Component analysis research highlights the essential role of this module, showing that DBT interventions including skills training are more effective than those without it in reducing NSSI and alleviating co-occurring depression and anxiety (Linehan et al., 2015). This structured, psychoeducational group format has been successfully adapted across contexts. For example, a pilot study of a condensed, DBT-based parental resilience and skills training program for families of adolescents with cancer reported feasibility and was associated with reduced short-term NSSI recurrence and improved adolescent psychological symptoms (Huang et al., 2025). Delivered in a weekly group format, DBT-ST provides systematic instruction in these skill sets, aiming to equip individuals with adaptive alternatives to maladaptive behaviors like NSSI.

### 4.3 Neurobiological correlates and predictors of DBT response

An emerging line of inquiry aims to elucidate the neurobiological mechanisms underlying the therapeutic effects of DBT. Neuroimaging studies are increasingly mapping the neuroplastic changes associated with this and similar skills-based interventions, which may explain improvements in emotion regulation.

Foundational evidence for DBT-induced neuroplasticity comes from a pivotal fMRI study by Goodman (Goodman et al., 2014). In unmedicated patients with Borderline Personality Disorder (BPD), 12 months of standard DBT led to significant reductions in amygdala hyperactivity and improved amygdala habituation to emotional stimuli, changes that were correlated with self-reported improvements in emotion regulation. This finding supports the hypothesis that DBT directly targets limbic hyperreactivity, a core neural substrate of affective instability.

Beyond examining post-treatment changes, research is exploring pre-treatment neural signatures as predictors of therapeutic response. Although direct neuroimaging predictors for DBT response in NSSI are still emerging, a comprehensive meta-analysis of treatment effects in BPD and related disorders indicates that psychotherapy, including DBT-like interventions, consistently induces activation changes in frontal regions (e.g., middle frontal gyrus) implicated in top-down control (Luo et al., 2023). This suggests that the functional integrity of prefrontal circuits may be a key determinant of treatment efficacy. Furthermore, mechanism-based psychotherapy research in BPD has demonstrated that successful treatment (e.g., anti-aggression therapy) can not only reduce amygdala activation but also enhance functional connectivity between the amygdala and the dorsomedial prefrontal cortex (Neukel et al., 2021), providing a direct model for how therapy may strengthen fronto-limbic communication.

Reviews synthesizing this growing field affirm these patterns. A dedicated review of neural changes after DBT in BPD concluded that treatment is associated with deactivation of the amygdala and anterior cingulate cortex, alongside modulations in inferior frontal gyrus activity related to inhibitory control (Iskric and Barkley-Levenson, 2021). A broader “State of the Science” review on DBT acknowledges the treatment’s efficacy and underscores the need for more research into its mechanisms, including neurobiological predictors (Rizvi et al., 2024).

Cumulatively, the evidence converges on a model where DBT aids in normalizing dysfunctional fronto-limbic circuitry through interconnected processes: (1) regulating bottom-up limbic reactivity (e.g., amygdala); (2) enhancing top-down prefrontal control (e.g., dlPFC, vlPFC, ACC); and (3) fostering more adaptive functional connectivity between these systems. Preliminary studies on other psychotherapies for BPD further support the centrality of improved brain connectivity, such as hippocampal networks, in symptom reduction (Aydin et al., 2025).

In summary, evidence from task-based activation, connectivity, and meta-analytic studies indicates that DBT and related skills-based therapies promote neuroplastic changes within the emotion regulation network. By cultivating skills that engage and strengthen prefrontal inhibitory pathways while modulating limbic hyperreactivity, these therapies can help individuals with NSSI disrupt the automatic cycle of self-injury and develop more sustainable, cognitively-mediated coping strategies.

## 5 Risk and protective factors

Empirically identified risk factors for the development of NSSI with addictive characteristics are multifaceted, encompassing developmental, cognitive, interpersonal, and psychiatric domains. A seminal systematic review and meta-analysis demonstrated that childhood maltreatment, particularly emotional abuse, constitutes a significant risk factor for NSSI (Liu et al., 2018). The adverse effects of early adversity may be mediated through pathways such as depressive symptoms, with individual differences in cognitive reappraisal and emotional reactivity serving as moderators (Gong and Zhang, 2024). Longitudinal birth cohort studies further identify early childhood predictors, revealing that parental factors at age six—specifically parenting stress and parental negativity/hostility—significantly forecast NSSI engagement during adolescence, even when controlling for child temperament and external events (Wichstrom and Wichstrom, 2024).

At the cognitive level, meta-analytic evidence establishes a positive correlation between rumination—encompassing its brooding and depressive subtypes—and NSSI, indicating a shared vulnerability in maladaptive emotion regulation processes (Cheung et al., 2024).

In addition to family dynamics, the interpersonal and intrapersonal environment is crucial for understanding NSSI risk and resilience. Negative family processes, such as parental psychological control, directly increase the risk for NSSI. This relationship is serially mediated by increased parent-related loneliness and subsequent depressive symptoms, forming a clear pathway from maladaptive parenting to self-harm (Guo et al., 2022). The detrimental impact of such family and interpersonal stressors converges on core psychological vulnerabilities. Longitudinal research confirms that psychological distress and rumination share bidirectional, reinforcing relationships with NSSI over time, creating a sustained maladaptive cycle (Buelens et al., 2019). Furthermore, the link between broader childhood adversity (e.g., maltreatment) and NSSI severity is significantly mediated by difficulties in emotion regulation and depressive symptoms, with emotion dysregulation often being the stronger pathway (Hu et al., 2023). Crucially, individual differences in cognitive and emotional resources can buffer these risks. Regulatory emotional self-efficacy (one’s belief in managing emotions) moderates the aforementioned pathway from parental psychological control to NSSI, highlighting a key protective factor (Guo et al., 2022). Similarly, trait hope—particularly the belief in finding pathways to goals—can attenuate the association between depressive symptoms and NSSI, especially among female adolescents (Jiang et al., 2018). Moreover, peer influence is a critical social determinant of NSSI, operating not only through behavioral contagion but, more significantly, through the experience of peer victimization. Longitudinal and cross-sectional studies consistently identify both traditional peer victimization and cybervictimization as potent risk factors for NSSI (Li et al., 2024; Ruan et al., 2024; Xu M. et al., 2025; Xu Y. et al., 2025). The psychological pathways linking victimization to self-harm are well-articulated. Victimization can lead to NSSI by eroding self-esteem and fostering alexithymia (difficulty identifying and describing feelings), with these two factors often acting in a chain to mediate the relationship (Guo et al., 2024). This process is exacerbated by the frustration of core interpersonal needs, such as feeling like a burden on others (perceived burdensomeness) (Li et al., 2024). Furthermore, the distress caused by victimization frequently translates into depressive symptoms, which in turn drive NSSI (Xu M. et al., 2025; Xu Y. et al., 2025). Individual traits critically moderate these risk pathways. Low self-esteem not only acts as a mediator but also amplifies the risk by strengthening the links between social anxiety, impulsivity, and NSSI (Zou et al., 2024). Conversely, psychological resilience serves as a key protective factor, directly mitigating NSSI risk and buffering the negative impact of alexithymia (Zhang et al., 2023). When resilience is low and loneliness is high, the path from emotional deficits to self-harm becomes particularly pronounced (Zhang et al., 2023). In summary, peer influence on NSSI is best understood as a network of social stressors (victimization) that trigger identifiable cognitive-emotional vulnerabilities (low self-esteem, alexithymia, depressive symptoms, thwarted belongingness). The transition from experiencing these vulnerabilities to engaging in self-harm is powerfully moderated by the presence or absence of protective traits such as resilience and healthy self-esteem.

In summary, the development of NSSI with addictive features is best understood through an integrated diathesis-stress and resilience framework. This model posits that early vulnerabilities—established through childhood adversity and maladaptive family dynamics—create a foundation of emotional dysregulation and negative self-schema. These latent vulnerabilities are subsequently activated by proximal interpersonal stressors, particularly peer victimization, which trigger and reinforce maladaptive cognitive-emotional processes such as rumination, alexithymia, and acute distress. The act of NSSI then functions as a potent, albeit harmful, short-circuit within this escalating cycle of affective and social pain. Crucially, this entire pathway can be attenuated or disrupted by key protective factors, including psychological resilience, regulatory self-efficacy, and trait hope. Therefore, effective prevention and intervention must adopt a multi-systemic approach that concurrently mitigates developmental and social risks while proactively cultivating individual capacities for emotion regulation and adaptive coping.

## 6 Conclusion and future directions

In conclusion, accumulating evidence endorses the applicability of an addiction framework in comprehending severe, repetitive NSSI. This viewpoint is reinforced by common behavioral characteristics (such as craving and loss of control), a neurobiological foundation involving dysfunctional fronto-limbic reward and regulatory circuits, and a developmental trajectory where initial vulnerabilities and interpersonal stressors interact to cultivate a maladaptive cycle of distress and immediate, self-injurious relief. DBT currently stands as the most efficacious intervention, likely owing to its ability to enhance top-down emotional regulation.

To propel the field forward and address the inherent limitations of the current model—notably its reliance on cross-sectional neuroimaging and data from severe clinical populations—forthcoming endeavors should prioritize the following: (1) Precision Phenotyping: Identifying neurobehavioral indicators that forecast the transition from sporadic to addictive NSSI, facilitating earlier and more precise intervention. This includes validating the proposed addictive subtype in broader, community-based samples. (2) Mechanism-Targeted Treatments: Formulating and evaluating innovative interventions that directly target the fundamental reinforcing neurocircuitry of addictive NSSI, surpassing current psychotherapeutic methods. (3) Integrated Care Models: Implementing phased, multi-systemic strategies that integrate evidence-based psychotherapy with family/peer support and appropriate biomedical treatments, customized to the individual’s disease stage and specific risk profile. Embracing this integrated model—which intertwines addictive behavior, neurobiology, and developmental context—is imperative for transcending symptomatic control and progressing towards disrupting the entrenched cycle of self-injurious addiction. (4) Future work must address key conceptual challenges to the addiction model. These include: (a) reconciling the model with the common “maturing out” of NSSI in late adolescence, potentially by identifying subtypes more prone to persistence; (b) employing longitudinal designs to move beyond correlational neuroimaging evidence; and (c) testing the model’s premises in community-based samples to clarify its generalizability beyond severe clinical populations.
